# Evolution of Cardiogenic Shock Management and Development of a Multidisciplinary Team-Based Approach: Ten Years Experience of a Single Center

**DOI:** 10.3390/jcm13072101

**Published:** 2024-04-03

**Authors:** Leonardo Belfioretti, Matteo Francioni, Ilaria Battistoni, Luca Angelini, Maria Vittoria Matassini, Giulia Pongetti, Matilda Shkoza, Luca Piangerelli, Tommaso Piva, Elisa Nicolini, Alessandro Maolo, Andi Muçaj, Paolo Compagnucci, Christopher Munch, Antonio Dello Russo, Marco Di Eusanio, Marco Marini

**Affiliations:** 1 Intensive Care Unit, Cardiology Department, Azienda Ospedaliero-Universitaria delle Marche, 60126 Ancona, Italy; matteo.francioni@ospedaliriuniti.marche.it (M.F.); ilaria.battistoni@ospedaliriuniti.marche.it (I.B.); mariavittoria.matassini@ospedaliriuniti.marche.it (M.V.M.); marco.marini@ospedaliriuniti.marche.it (M.M.); 2 Intervention Cardiology, Azienda Ospedaliero-Universitaria delle Marche, 60126 Ancona, Italy; tommaso.piva@ospedaliriuniti.marche.it (T.P.); elisa.nicolini@ospedaliriuniti.marche.it (E.N.);; 3 Cardiology and Arrhythmology Clinic, Azienda Ospedaliero-Universitaria delle Marche, 60126 Ancona, Italy; paolocompagnucci1@gmail.com (P.C.); antonio.dellorusso@ospedaliriuniti.marche.it (A.D.R.); 4 Cardiac Anaesthesia and Intensive Care Unit, Azienda Ospedaliero-Universitaria delle Marche, 60126 Ancona, Italy; christopher.munch@ospedaliriuniti.marche.it; 5 Cardiac Surgery Unit, Azienda Ospedaliero-Universitaria delle Marche, 60126 Ancona, Italy; marco.dieusanio@ospedaliriuniti.marche.it

**Keywords:** cardiogenic shock, acute cardiovascular syndrome, acute heart failure, multidisciplinary team, mechanical cardiac support

## Abstract

**Background:** The management of cardiogenic shock (CS) after ACS has evolved over time, and the development of a multidisciplinary team-based approach has been shown to improve outcomes, although mortality remains high. **Methods:** All consecutive patients with ACS-CS admitted at our CICU from March 2012 to July 2021 were included in this single-center retrospective study. In 2019, we established a “shock team” consisting of a cardiac intensivist, an interventional cardiologist, an anesthetist, and a cardiac surgeon. The primary outcome was in-hospital mortality. **Results:** We included 167 patients [males 67%; age 71 (61–80) years] with ischemic CS. The proportion of SCAI shock stages from A to E were 3.6%, 6.6%, 69.4%, 9.6%, and 10.8%, respectively, with a mean baseline serum lactate of 5.2 (3.1–8.8) mmol/L. Sixty-six percent of patients had severe LV dysfunction, and 76.1% needed ≥ 1 inotropic drug. Mechanical cardiac support (MCS) was pursued in 91.1% [65% IABP, 23% Impella CP, 4% VA-ECMO]. From March 2012 to July 2021, we observed a significative temporal trend in mortality reduction from 57% to 29% (OR = 0.90, *p* = 0.0015). Over time, CS management has changed, with a significant increase in Impella catheter use (*p* = 0.0005) and a greater use of dobutamine and levosimendan (*p* = 0.015 and *p* = 0.0001) as inotropic support. In-hospital mortality varied across SCAI shock stages, and the SCAI E profile was associated with a poor prognosis regardless of patient age (OR 28.50, *p* = 0.039). **Conclusions:** The temporal trend mortality reduction in CS patients is multifactorial, and it could be explained by the multidisciplinary care developed over the years.

## 1. Introduction

Cardiogenic shock (CS) following acute myocardial infarction (AMI) is still the leading cause of death in patients with ischemic cardiomyopathy and represents the most severe form of acute heart failure (AHF) syndromes [1]. The pathophysiology of CS after AMI is characterized by an acute reduction in cardiac output despite the presence of adequate intravascular volume, leading to severe peripheral hypoperfusion associated with tissue hypoxia and increased serum lactate levels. The evaluation of intravascular volume and fluid responsiveness is challenging in critically ill patients, and it is particularly crucial, as over-resuscitation could produce significant morbidity and mortality [2]. Considering the heterogeneity of CS patients and given the limitations of the various predictors of fluid responsiveness, a multimodal tailored approach is advisable, including static and dynamic indices along with echocardiographic assessment and dynamic tests [3]. Myocardial revascularization, inotropic drug therapies, and mechanical assistance devices represent the main elements to interrupt negative event spirals leading to multiorgan failure [4]. However, only primary percutaneous coronary intervention (primary-PCI) has been shown to reduce mortality in CS following AMI, while neither intra-aortic balloon pumps (IABPs) nor microaxial pumps have demonstrated an impact on survival endpoints [5,6,7]. Risk stratification appears crucial for the early identification of high-risk patients who need rapid mechanical cardiac support (MCS) implementation, and at the same time, it allows clinicians to avoid futile procedures. The recent Society for Cardiovascular Angiography and Interventions (SCAI) shock stage classification, along with its update with the three-axis model of CS, has been proposed to better define shock severity and help clinicians to identify early phases [8]. Indeed, current studies have confirmed the correlation of the SCAI staging system with mortality across all subgroups of ACS patients [9]. The incidence of ischemic CS complicating ACS has increased in the last decade, and most recent registries have documented an improvement in in-hospital outcomes with a mortality rate near 40–50% [10]. The overall reduction in in-hospital mortality appears to be multifactorial, caused by an early revascularization strategy, an improvement in MCS management, and the introduction of a team-based approach to CS. Recent data suggest that a multidisciplinary “Heart Team” applied to CS care could produce survival benefits and reduce patients’ morbidity [11,12]. The aims of the present study are: (I) to examine the ten years’ temporal trends of mortality in a tertiary-level cardiac intensive care unit (CICU), (II) to analyze changes in MCS use, inotropic drug therapy, and patient characteristics admitted over time, (III) to evaluate the prognostic insight of a SCAI staging system in our sample, and (IV) to describe in-hospital mortality predictors in a real-world cohort of patients with ischemic CS.

## 2. Materials and Methods

### 2.1. Patient Enrollment

All consecutive patients > 18 years with CS after AMI admitted at our tertiary-level CICU at “Azienda Ospedaliero-universitaria delle Marche” (Ancona, Italy) from March 2012 to July 2021 were included in this single-center retrospective study. Our analysis also included patients with acute decompensated heart failure (ADHF) complicated by CS. Exclusion criteria were: (I) non-ischemic CS, (II) post-surgery CS, and (III) all subjects who died prematurely upon arrival at CICU or in catheterization laboratory within 8 h.

### 2.2. Definitions

We defined acute myocardial infarction (AMI) according to the criteria of “Fourth Universal Definition of myocardial infarction”, i.e., at least one value of high-sensitive cardiac troponin (hs-cTn) increased above the 99th percentile upper reference limit (URL) associated with one of the following: symptoms compatible with myocardial ischemia, appearance of pathological Q waves, ST segment variations or T-wave abnormalities on electrocardiogram (ECG), and imaging alterations in a pattern consistent with an ischemic etiology [13].

The diagnosis of CS was performed at CICU admission, evaluating clinical signs and symptoms of peripheral hypoperfusion (cold extremities, reduced urine output < 30 mL/h, mental confusion, dizziness, narrow pulse pressure), with the presence of hypotension defined as systolic blood pressure (SBP) < 90 mmHg for more than 30 min or the need of catecholamines to maintain SBP > 90 mmHg and serum lactate > 2 mmol/L [1].

The vasoactive inotropic score was calculated using the following formula: VIS = dopamine (μg/kg/min) + dobutamine (μg/kg/min) + 100 × epinephrine (μg/kg/min) + 100 × norepinephrine (μg/kg/min) + 10 × milrinone (μg/kg/min) + 10,000 × vasopressin (units/kg/min) + 50 × levosimendan (μg/kg/min) [14].

### 2.3. Data Collection

Data were retrospectively obtained from electronic medical records and collected anonymously in a database. Routine data recording included demographics, cardiovascular risk factors, previous medical history, echocardiographic data, pharmacological and ventilatory therapy performed, use of mechanical cardiac support (MCS) and/or continuous renal replacement therapy (CRRT), as well as laboratory data. The outcome of coronary angiography study and primary PCI were included, and we also applied in-hospital mortality risk scores such as CardShock risk score [15]. Moreover, to quantify the extent of pharmacological cardiovascular support, we used the vasoactive inotropic score (VIS). Based on more recent position statement, SCAI classification criteria were retrospectively applied, and each CS patient was assigned to one of the five subgroups (from A = “At risk”, B = “Beginning”, C = “Classic”, D = “Deteriorating”, to E = “Extremis”) at CICU admission [8]. In 2019, we established a “shock team” consisting of cardiac intensivist, interventional cardiologist, anesthetist, and cardiac surgeon. CS patients were managed according to the expertise of the center and in accordance with current guidelines. The primary outcome was all-cause in-hospital mortality.

### 2.4. Statistical Analysis

Continuous variables were checked for normality using the Shapiro–Wilk test and were reported as mean and standard deviation if normally distributed or as median and interquartile range (IQR) if non-normally distributed. The association of clinical, echocardiographic, and laboratory parameters with the primary outcome was assessed with logistic regression. Comparisons between groups were performed with Student’s *t*-test for normally distributed variables or Wilcoxon’s rank sum test for non-normally distributed variables. A two-sided *p* < 0.05 defined statistical significance. All statistical analyses were performed with the Software R version 4.2.0 (22 April 2022) (R Foundation for Statistical Computing, Vien, Austria).

## 3. Results

### 3.1. Baseline Characteristics, Temporal Trend of In-Hospital Mortality, and Predictors of Poor Outcome

From March 2012 to July 2021, we enrolled 167 patients with ischemic CS, of which 67% were male. The shock etiology was STEMI in 84% of cases, N-STEMI in 11%, and ADHF in 5%. The average age was 71 years (61–80). Patients had severe LV dysfunction in 66%, and the baseline mean serum lactate was 5.2 (3.1–8.8) mmol/L. The mean CardShock risk score at admission was 6 (5–7). Twenty-two percent of patients were resuscitated from out-of-hospital cardiac arrest. All patients required inotropes, and 76.1% needed ≥ 1 inotropic drug. Seventy-one percent required dopamine [mean dose 5.6 (2.4–11.3) mcg/kg/min], 65% required noradrenaline [mean dose 0.10 (0.05–0.18) mcg/kg/min], 32% required dobutamine [mean dose 4.5 (2.2–15.9) mcg/kg/min], and 17.4% received levosimendan alone [mean dose 0.1 mcg/kg/min]. Mechanical cardiac support (MCS) was pursued in 91.1% [65% IABP, 23% Impella CP, 4% VA-ECMO]. Invasive mechanical ventilation was required in 63% of cases. All patients underwent coronary angiography, and 81% needed primary PCI. In STEMI patients, the mean time before primary PCI was 125 min. Table 1 summarizes the baseline characteristics at the admission.

**Figure 1:**
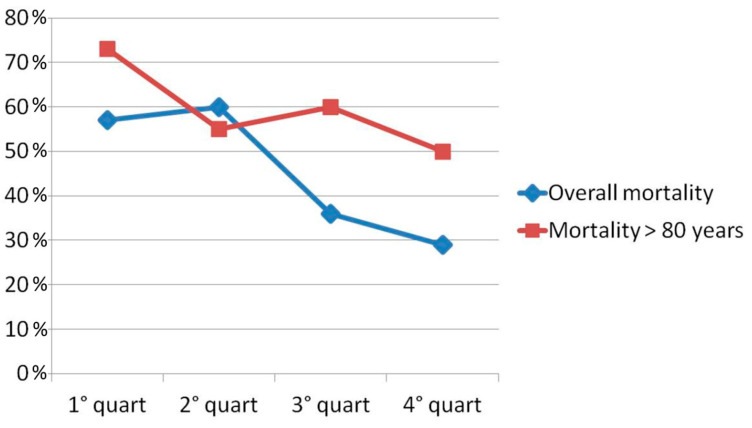
Temporal trend mortality reduction from first time quartile to fourth quartile in overall population and in patients > 80 years. (First quartile: 7 March 2012–12 July 2014; Second quartile: 12 July 2014–4 April 2017; Third quartile: 4 April 2017–19 August 2019; Fourth quartile: 19 August 2019–3 July 2021).

The overall rate of in-hospital mortality in our sample was 46%. The trends analysis showed a significant decrease in in-hospital mortality from 57% in the first time quartile (7 March 2012–12 July 2014) to 29% in the fourth quartile (19 August 2019–3 July 2021) (OR 0.90, 95%CI 0.84–0.96, *p* = 0.0015; Figure 1). The mortality in patients aged 80 or more (age ≥80 years; *n* = 42) was higher than that in patients ˂ 80 years, and the association of the quartile of admission with in-hospital death was no more statistically significant (OR 0.75, 95%CI 0.402–1.379, *p* = 0.361). Also, by dividing the sub-population of patients aged 80 or more (*n* = 42) into four new quartiles, the association of the quartile of admission with in-hospital death was not statistically significant (OR = 0.77, 95%CI = 0.429–1.336, *p* = 0.355). The multivariate analysis showed that age > 64 years (OR 1.36, 95%IC 1.04–1.79, *p* = 0.029), lactate level at admission (OR 1.03, 95%CI 1.01–1.06, *p* = 0.015), left ventricular ejection fraction (EF) at baseline (OR 0.99, 95%CI 0.98–0.99, *p* = 0.047), and the presence of three-vessel coronary artery disease (OR 0.75, 95%CI 0.59–0.95, *p* = 0.019) were in-hospital mortality predictors.

### 3.2. SCAI Stage System ANALYSIS and Changing of CS Patients Baseline Characteristics Over Time

SCAI classification criteria were retrospectively applied, and each CS patient was assigned to one of the five subgroups at CICU admission. The proportion of SCAI shock stages from A to E were 3.6%, 6.6%, 69.4%, 9.6%, and 10.8%, respectively. The distribution of clinical and laboratory parameters, as well as inotropic drug therapies, ventilatory support, and dialysis, changed across SCAI stages. The SCAI E profile showed the worst systemic hypoperfusion parameters, such as high arterial baseline lactate (value 11.3 mmol/mol (8.3–15.9), *p* <0.001) and low pH values (value 7.12 (7.05–7.21), *p* 0.002). SCAI E patients required higher inotropic drug support (mean VIS value 54.0 (26.5–71.4), *p* = 0.001), 94% underwent mechanical ventilation (*p* = 0.008), CRRT was not pursued in these patients compared to other SCAI stages (*p* = 0.017), and the length of stay in CICU was very short (average stay was 1 day, *p* < 0.001). Moreover, there was a significant variation in plasmatic procalcitonin values between the B and C stages versus the D and E stages (*p* = 0.037). We also observed a trend towards lower mean arterial pressure values in SCAI D and E patients (58 mmHg (51–74) and 50 mmHg (42–57), respectively, versus SCAI C 67 mmHg (58–74)) and higher plasmatic high-sensitivity troponin values compared to SCAI C profile (200.00 ng/mL (112.15–200.00) and 154.00 ng/mL (7.67–200.00) versus SCAI C 117.00 ng/mL (20.40–200.00)). The proportion of out-of-hospital cardiac arrest was higher in the SCAI D and E stages compared to SCAI B and C, although not statistically significant (SCAI D and E 38% and 22%, respectively, versus SCAI B 9% and C 20%, *p* = 0.365). The patients’ baseline characteristics, the etiology of CS, and the mechanical support pursued did not change across SCAI profiles. Table 2 summarizes the characteristics of patients according to SCAI classification.

The in-hospital mortality varied across SCAI shock stages: 50% in SCAI A (three deaths), 36% in SCAI B (OR 0.52, 95% CI 0.10–4.33, *p* = 0.441), 23% in SCAI C (OR 0.40, 95% CI 0.10–2.73, *p* = 0.187), 88% in SCAI D (OR 4.99, 95% CI 0.85–29.42, *p* = 0.076), and 100% in SCAI E (OR 46.60, 95% CI 1.79–1213.80, *p* = 0.021). The association of stage E with in-hospital death was independent of patient age at multivariable analysis (OR 28.50, *p* = 0.039), as shown in Table 3. All the deaths that we observed in the SCAI A stage (three deaths) were due to the overlap of septic components on CS, as evidenced by the high mean values of plasmatic procalcitonin in this subgroup.

Over time, we observed a significant change in CS patients’ baseline characteristics. From the first time quartile to the fourth time quartile, there was a decrease in the mean age of patients admitted for ischemic CS from 78 ± 15 to 65 ± 14 years (*p-trend* 0.0002). The patients admitted also showed an overall improved kidney function with an eGFR at hospitalization of 45 ± 23 mL/min/m^2^ to 60 ± 39 mL/min/m^2^ (*p-trend* 0.002). We applied at baseline the CardShock risk score, and we found a significant decrease in the mean score from 6 ± 2 to 5 ± 2 ((*p-trend* 0.002). The main cardiovascular risk factors, such as arterial hypertension, diabetes mellitus, and dyslipidemia, did not change over time (*p-trends*, respectively, 0.33, 0.10, and 0.14). Table 4 summarizes changes in baseline characteristics over time.

### 3.3. Trends in MCS Use and Inotropic Drug Therapy Management

From 2012 to 2021, there was a significant change in the use of MCS and different management of inotropic drug therapy in CS patients. In the first time quartile, IABP was the main MCS in our center, with a utilization rate of 98%. However, we found a significant decrease in IABP use (*p-trend* 0.01) and, at the same time, an increase in microaxial pump implants (Impella CP) with a *p-trend* of 0.0005. In particular, Impella CP catheter use rose from only 2% in 2012–2014 to 50% in the last time quartile (2019–2021, Figure 2). The management of inotropic support therapies in CS patients changed over time, with a significant increase in drugs with inodilator action, such as dobutamine and levosimendan (*p-trends*, respectively, 0.015 and 0.0001), and a consequent decrease in dopamine and adrenaline administration (*p-trends*, respectively, 0.0007 and 0.0008).

**Figure 3:**
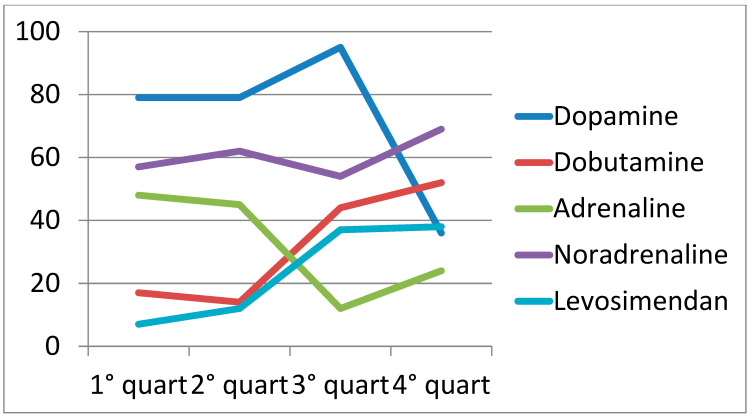
Change in management of inotropic drug therapies over time. (First quartile: 7 March 2012–12 July 2014; Second quartile: 12 July 2014–4 April 2017; Third quartile: 4 April 2017–19 August 2019; Foutth quartile: 19 August 2019–3 July 2021).

We did not observe a trend in noradrenaline administration, which remains the first-choice vasopressor (*p-trend* 0.40) (Figure 3).

Trend analysis also showed a decrease in the amount of pharmacological cardiovascular support quantified by applying the VIS formula. In detail, the level of inotropic pharmacological assistance dropped by about 10 points in the last decade, from 22 points (2012–2014) to 13 points (2019–2021), with a *p-trend* of 0.0003.

## 4. Discussion

In this retrospective 10-year study, we found a significant temporal trend in mortality reduction, particularly the in-hospital mortality reduced from 57% to 29%. This finding aligns with existing scientific literature that demonstrates a consistent, albeit slight, reduction in overall CS mortality rates [16], although this trend has not been universally confirmed by all studies [17]. For instance, Shah et al. reported an increased mortality rate across all CS hospitalizations in the United States from 2005 to 2014, regardless of AMI status. However, it is important to note that these results may be influenced by the inclusion of non-ischemic CS patients and heterogeneous data from peripheral hospitals.

Age was identified as a predictor of mortality in our sample as well. Patients older than 80 years exhibited a mortality rate exceeding 55–60%, making them an extremely vulnerable subgroup burdened by high comorbidities. This often contraindicates the use of MCS and complete revascularization. Given the exceptionally high mortality rate in our center, the MCS of choice for this subgroup is typically IABP when feasible. Conversely, microaxial pumps do not appear to be a cost-effective solution for this demographic [18]. While age correlates with increased mortality independent of shock severity, it is important to note that age alone should not be considered an absolute contraindication to MCS use. Additionally, the treatment of very elderly patients may also be influenced by local resources and hospital management policies.

We observed a shift in the characteristics of the admitted population over time, noting a significant trend in age, glomerular filtration rate, and CardShock mean score. However, we did not observe any significant changes in the overall cardiovascular risk profile. The increasing hospitalization of younger patients over time, who exhibit a lower degree of chronic renal failure, correlated with a decreasing mean CardShock score. Specifically, the score decreased from 6 ± 2 to 5 ± 2, indicating a corresponding risk of in-hospital mortality of >70% and 30–45%, respectively [15]. We hypothesize that this trend is linked to improvements in the acute myocardial infarction network [19] and increased awareness regarding the crucial importance of timely and accurate CS diagnosis, particularly in emergency departments and peripheral centers.

Over time, there have been notable changes in the selection and management of MCS. Initially, in the first quartile, IABP was the exclusive MCS used in our center, with an implantation rate of 98%. However, following the publication of the IABP-SHOCK trial in 2012 [20] and the introduction of microaxial pumps, there has been a significant shift in MCS utilization. This change has resulted in a decline in IABP use and a subsequent rise in Impella CP implantations. Nonetheless, IABP continues to hold a significant share of MCS in our CICU, with an implantation rate of 50% in 2021.

Despite being a low-cost and readily available device, making it a viable option for older patients (>80 years), particularly during the early stages of CS, the IABP is only given a third-class indication in current guidelines [21]. Yet, its usage remains prevalent in Italian CICUs [22]. Furthermore, IABP is still recommended for AMI mechanical complications, which continue to affect 1–2% of hospitalizations for ischemic CS [21].

The introduction of microaxial pumps has changed daily clinical practice. These pumps offer superior hemodynamic performance for treating unstable patients and allow for more comprehensive revascularization procedures [23]. Additionally, microaxial pumps enable a reduction in the dosage of inotropic drug therapies, potentially explaining the observed decrease in the VIS score. Increased awareness regarding the appropriate use of inotropic drugs, coupled with the choice of administering inodilator drugs at the lowest effective dosage, has contributed to minimizing side effects such as ventricular arrhythmias. This is particularly crucial, as these arrhythmias can be life-threatening in patients with CS. Dopamine was still administered in approximately 30% of patients in the fourth time quartile (2019–2021). This inotropic drug is used primarily in catheterization laboratories and in some spoke centers, although its use has been steadily decreasing.

SCAI shock stage classification is useful for identifying early CS phases, and it helps to stratify patients’ mortality risk. In our study sample, we observed a strong association between the SCAI E profile and poor prognosis, with a 100% in-hospital mortality rate [24]. Patients classified as SCAI E exhibited hemodynamic parameters that indicated profound systemic hypoperfusion, including markedly elevated lactate levels (mean value of 11.3 mmol/mol (8.3–15.9)) and low pH levels (mean value of 7.12 (7.05–7.21)). These patients required high dosages of inotropic drugs and a high rate of mechanical ventilation. Continuous renal replacement therapy (CRRT) was generally not pursued for these patients, and their length of stay in the CICU was notably short, reflecting the severe hemodynamic deterioration they experienced. The early recognition of refractory shock is paramount for minimizing the “door-to-support time” [25] and promptly initiating high-performance MCS, such as Impella or VA-ECMO. In our study, we found varying in-hospital mortality rates across different SCAI stages. Consistent with previous research, we noted a slight increase in in-hospital mortality among SCAI B patients compared to SCAI C patients (36% versus 32%, respectively). This observation may be attributed to the challenges associated with identifying patients in the early stages of shock who present with peripheral hypoperfusion but maintain normal blood pressure values. The SCAI B profile identifies patients with normotensive hypoperfusion and normal arterial lactate levels who are at risk of being under-recognized and consequently undertreated [26].

Finally, we postulate that the temporal trend mortality reduction observed in our sample is multifactorial. First, the change in MCS management plays a key role; in particular, the Impella CP catheter provides greater hemodynamic support than IABP, allowing left ventricular unloading, an increase in mean arterial pressure, and a consistent improvement in end-organ perfusion. However, despite the undoubted advantages in pathophysiological terms, the trials that evaluated the efficacy and safety of microaxial pumps gave inconclusive results [27,28]. In this context, the patient selection and timing of MCS initiation appear crucial [29]. Since 2019 in our CICU, the Impella CP catheter has been routinely implanted before PCI in CS patients, in accordance with data from the USpella registry that have shown an improvement in in-hospital survival in pre-PCI subgroups with more complete myocardial revascularization [30]. The improvement of the STEMI network with the early centralization of younger patients and the systematic application of the SCAI staging system at the time of admission has played a crucial role in the reduction of mortality. Over the years, our center has adopted a multidisciplinary approach to managing patients with ischemic CS. We believe that this integrated approach has contributed to the observed improvement in in-hospital outcomes, consistent with the survival benefits reported in recent studies [31]. Our hospital’s multidisciplinary CS team consists of a cardiac intensivist, an anesthetist, an interventional cardiologist, and a cardiac surgeon. This specialized “shock team” plays a crucial role in identifying patients who may benefit from mechanical circulatory support, thereby avoiding unnecessary procedures.

Additionally, the CS team’s role extends beyond the MCS therapies [32] and revascularization strategies, including the titration and choice of inotropic drug therapies, the management of ventilatory support including mechanical ventilation, the nutritional aspects of critical patients, and the use of renal replacement therapy when indicated.

## 5. Study Limitations

This study has several limitations that should be acknowledged. First, its retrospective observational design may introduce selection bias and confounding factors that cannot be fully controlled for. Additionally, it is a single-center study, and the sample size is relatively small, which limits the generalizability of our findings. Moreover, since our center exclusively manages VA-ECMO in the cardiac surgery intensive care unit, our results may not be applicable to centers with different management protocols or patients with non-ischemic CS. These limitations should be considered when interpreting the results of this study.

## 6. Conclusions

The observed temporal trend in mortality reduction over the past decade in our CICU is multifactorial, stemming from changes in baseline patient characteristics upon admission, the increased utilization of high-performance MCS, modified inotropic drug management, and heightened awareness regarding the significance of timely and accurate CS diagnosis. The application of the SCAI shock staging system is advised for early shock phase identification, serving as a dependable tool for mortality risk stratification. Notably, the SCAI E profile is linked to a particularly dismal prognosis, regardless of the interventions employed. The multidisciplinary care approach to CS that has evolved over the years encompasses all facets of shock management and has the potential to enhance patient survival. Nevertheless, prospective randomized trials are imperative to further elucidate the genuine clinical impact and cost-effectiveness of a standardized, team-based approach in managing ischemic CS.

## Figures and Tables

**Figure 2:**
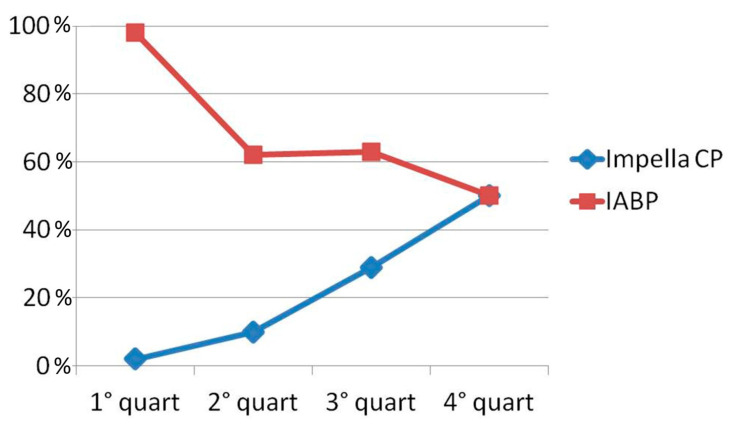
Temporal trend in Impella CP and IABP use. (First quartile: 7 March 2012–12 July 2014; Second quartile: 12 July 2014–4 April 2017; Third quartile: 4 April 2017–19 August 2019; Fourth quartile: 19 August 2019–3 July 2021).

**Table 1:** Baseline characteristics at the admission. BMI: body mass index; PCI: percutaneous coronary intervention; CABG: coronary artery bypass graft; TIA: transient ischemic attack; COPD: chronic obstructive pulmonary disease; VSD: ventricular septal defect; eGFR: estimated glomerular filtration rate; C-PAP: continuous positive airway pressure; LVEF: left ventricular ejection fraction; IABP: intra-aortic balloon pump; ECMO: extracorporeal membrane oxygenation.

Baseline Characteristics	All Population (*n* = 167)
Age—years	71 (61–80)
Female sex—no. (%)	55 (33)
BMI—kg/m^2^	25.9 (23.7–28.6)
**Past medical history—no. (%)**	
Previous myocardial infarction	37 (22)
Prior PCI	27 (16)
Prior CABG	10 (6)
Hypertension	110 (66)
Diabetes	64 (38)
COPD	23 (14)
eGRF < 60 mL/min	99 (59)
Atrial fibrillation during hospitalization Preexistent permanenta atrial fibrillation	76 (46) 6 (4)
Stroke/TIA	16 (10)
Peripheral arterial disease	27 (16)
Smoker	70 (42)
**Baseline characteristics—2**	
Cause of cardiogenic shock—no (%):	
STEMI	140 (84)
NSTEMI	19 (11)
Acute decompensated heart failure	8 (5)
LVEF at admission—(%)	30 (22–35)
Right ventricle dysfunction—no. (%)	48 (29)
Moderate-severe mitral regurgitation—no. (%)	52 (31)
Severe aortic stenosis—no. (%)	3 (2)
CardShock score—no.	6 (5–7)
Major ACS complications—no. (%)	42 (25)
Resuscitated from cardiac arrest—no. (%)	36 (22)
VSD—no. (%)	3 (2)
Free wall rupture—no. (%)	2 (1)
Papillary muscle rupture—no. (%)	2 (1)
**Biochemistry**	
Arterial blood lactate at presentation (mmol/L)	5.2 (3.1–8.8)
eGFR at presentation (mL/min/1.73 m^2^)	49 (32–65)
**Mortality**	
In-hospital length stay, days	11 (4–19)
In-hospital mortality, *n* (%)	76 (46)
**Coronary angiography data—no. (%)**	
Coronary angiogram performed	160 (96)
Primary PCI	135 (81)
Single-vessel disease	47 (28)
Two-vessel disease	57 (34)
Three-vessel disease	55 (33)
Left main disease	27 (16)
**Mechanical cardiac support—no. (%)**	
IABP	103 (62)
Impella	37 (22)
ECMO/L-VAD	7 (4)
**Medications—no. (%)**	
Norepinephrine	109 (65)
Epinephrine	62 (37)
Dopamine	119 (71)
Dobutamine	54 (32)
Levosimendan	39 (23)
Vasoactive inotropic score—no.	15 (10–30)
Invasive mechanical ventilation—no. (%)	105 (63)
C-PAP—no. (%)	49 (29)

**Table 2:** The characteristics of patients according to SCAI classification. BMI: body mass index; PAD: peripheral arterial disease; COPD: chronic obstructive pulmonary disease; MI: myocardial infarction; eGFR: estimated glomerular filtration rate; C-PAP: continuous positive airway pressure; CRP: C-reactive protein; LVEF: left ventricular ejection fraction; IABP: intra-aortic balloon pump; ECMO: extracorporeal membrane oxygenation; CRRT: continuous renal replacement therapy; CICU: cardiac intensive care unit.

Variable	A (*n* = 6)	B (*n* = 11)	C (*n* = 116)	D (*n* = 16)	E (*n* = 18)	*p* Value
In-hospital death—*n* (%)	3 (50)	4 (36)	37 (32)	14 (88)	18 (100)	<0.001
Age (years)—median (IQR)	63 (56–69)	62 (52–78)	71 (62–79)	68 (62–80)	78 (65–82)	0.122
Female gender—no (%)	2 (33)	5 (46)	35 (30)	4 (25)	9 (50)	0.396
BMI (kg/m^2^)—median (Q1–Q3)	27.7 (25.9–30.4)	23.3 (22.3–25.9)	25.9 (23.9–29.2)	25.9 (24.9–27.5)	25.6 (24.2–27.0)	0.310
Arterial hypertension—*n* (%)	3 (50)	5 (46)	77 (66)	10 (63)	15 (83)	0.238
Dyslipidemia—*n* (%)	5 (83)	7 (64)	67 (58)	6 (38)	8 (4)	0.262
Diabetes mellitus—*n* (%)	1 (17)	2 (18)	46 (40)	4 (25)	11 (61)	0.091
Smoking—*n* (%)	2 (33)	3 (27)	52 (45)	7 (44)	6 (33)	0.755
PAD—*n* (%)	2 (33)	2 (18)	20 (17)	0 (0)	3 (17)	0.230
COPD—*n* (%)	0 (0)	0 (0)	22 (19)	1 (6)	0 (0)	0.083
Prior MI—*n* (%)	1 (17)	1 (9)	27 (23)	0 (0)	7 (39)	0.063
eGFR at hospitalization (mL/min)—median (IQR)	93 (53–121)	55 (35–102)	50 (32–67)	45 (29–57)	48 (29–53)	0.105
NSTEMI—*n* (%)	1 (17)	0 (0)	14 (12)	2 (13)	2 (11)	0.796
STEMI—*n* (%)	3 (50)	9 (82)	98 (85)	14 (88)	16 (89)	0.267
Out-of-hospital cardiac arrest—*n* (%)	2 (33)	1 (9)	23 (20)	6 (38)	4 (22)	0.365
Mechanical ventilation—*n* (%)	3 (50)	4 (36)	70 (60)	11 (69)	17 (94)	0.008
c-PAP—*n* (%)	1 (17)	6 (55)	36 (31)	4 (25)	2 (11)	0.140
MAP at hospitalization (mmHg)—median (Q1–Q3)	57 (57–61)	82 (69–85)	67 (58–74)	58 (51–74)	50 (42–57)	0.051
Heart rate (bpm)—median (Q1–Q3)	77 (70–90)	110 (100–135)	100 (85–112)	104 (95–110)	103 (93–112)	0.086
Arterial lactate at baseline (mg/dL)—median (Q1–Q3)	5.8 (3.6–7.9)	5 (3.0–7.6)	4.5 (2.6–7.7)	4.3 (3.7–6.3)	11.3 (8.3–15.9)	<0.001
Arterial pH at baseline—median (Q1–Q3)	7.29 (7.25–7.34)	7.38 (7.28–7.43)	7.29 (7.19–7.38)	7.26 (7.16–7.33)	7.12 (7.05–7.21)	0.002
Procalcitonin—median (Q1–Q3)	17.420 (7.867–72.475)	4.060 (0.530–5.235)	3.920 (0.820–10.900)	14.250 (9.055–31.675)	10.335 (6.702–13.967)	0.037
CRP (mg/dL)—median (Q1–Q3)	27.0 (23.5–28.2)	16.0 (11.7–20.1)	18.3 (11.1–25.5)	15.5 (6.7–20.5)	11.1 (7.0–12.7)	0.198
Troponin (ng/mL)—median (Q1–Q3)	31.60 (17.03–68.53)	54.40 (25.90–156.54)	117.00 (20.40–200.00)	200.00 (112.15–200.00)	154.00 (7.67–200.00)	0.051
LVEF (%)—median (Q1–Q3)	28 (25–38)	30 (25–33)	30 (23–35)	25 (20–35)	25 (20–30)	0.333
RV dysfunction—*n* (%)	3 (50)	0 (0)	32 (28)	7 (44)	6 (33)	0.063
IABP—*n* (%)	3 (50)	7 (64)	81 (70)	11 (69)	14 (78)	0.744
Impella—*n* (%)	3 (50)	3 (27)	25 (22)	2 (13)	2 (11)	0.269
ECMO—*n* (%)	1 (17)	1 (9)	3 (3)	2 (13)	0 (0)	0.065
CRRT—*n* (%)	3 (50)	3 (27)	37 (32)	5 (31)	0 (0)	0.017
Length of stay in CICU (days)—median (Q1–Q3)	32 (13–55)	17 (5–22)	12 (7–20)	4 (2–16)	1 (1–1)	<0.001
Vasoactive inotropic score—median (IQR)	15.1 (10.0–20.0)	15.0 (12.3–19.8)	13.8 (8.4–26.4)	28.0 (15.7–38.7)	54.0 (26.5–71.4)	0.001

**Table 3:** The association of the SCAI E stage with in-hospital death was independent of patient age at multivariable analysis.

Variable	OR	Lower CL	Upper CL	*p* Value
Age	1.05	1.02	1.08	0.004
SCAI B (compared to SCAI A)	0.47	0.09	2.64	0.394
SCAI C (compared to SCAI A)	0.28	0.07	1.13	0.07
SCAI D (compared to SCAI A)	3.85	0.63	23.34	0.143
SCAI E (compared to SCAI A)	28.50	1.19	685.91	0.039

**Table 4:** Change in patient characteristics at admission over time. (First quartile: 7 March 2012–12 July 2014; Second quartile: 12 July 2014–4 April 2017; Third quartile: 4 April 2017–19 August 2019; Fourth quartile: 19 August 2019–3 July 2021).

Variable	First Quartile of Time	Second Quartile of Time	Third Quartile of Time	Fourth Quartile of Time	*p-Trends*
Age—median (IQR)	78 (15)	72 (16)	69 (18)	65 (14)	0.0002
Arterial hypertension—*n* (%)	29 (69)	32 (76)	21 (51)	28 (67)	0.33
Dyslipidemia—*n* (%)	20 (48)	22 (52)	25 (61)	26 (62)	0.14
Diabetes mellitus—*n* (%)	20 (48)	16 (38)	16 (39)	12 (29)	0.10
CardShock score—median (IQR)	6 (2)	6 (2)	5.5 (4)	5 (2)	0.002
eGFR at hospitalization—median (IQR)	45 (23)	45 (33)	52 (48)	60 (39)	0.002
IABP—*n* (%)	41 (98)	26 (62)	26 (63)	23 (55)	0.01
Impella—*n* (%)	1 (2)	4 (10)	12 (29)	21 (50)	0.0005
Use of dopamine—*n* (%)	33 (79)	33 (79)	39 (95)	15 (36)	0.0007
Use of dobutamine—*n* (%)	7 (17)	6 (14)	18 (44)	22 (52)	0.015
Use of adrenaline—*n* (%)	20 (48)	19 (45)	5 (12)	10 (24)	0.0008
Use of noradrenaline—*n* (%)	24 (57)	26 (62)	22 (54)	29 (69)	0.40
Use of levosimendan—*n* (%)	3 (7)	5 (12)	15 (37)	16 (38)	0.0001
Vasoactive inotropic score—median (IQR)	22 (40)	21 (26)	12 (9)	13 (20)	0.0003

## Data Availability

The raw data supporting the conclusions of this article will be made available by the authors on request due to privacy restrictions.
